# Genetic Polymorphism of the Nrf2 Promoter Region (rs35652124) Is Associated with the Risk of Diabetic Foot Ulcers

**DOI:** 10.1155/2020/9825028

**Published:** 2020-08-15

**Authors:** Rajan Teena, Umapathy Dhamodharan, Daoud Ali, Kesavan Rajesh, Kunka Mohanram Ramkumar

**Affiliations:** ^1^Department of Biotechnology and SRM Research Institute, SRM Institute of Science and Technology, Kattankulathur, Tamil Nadu, India; ^2^Department of Zoology, College of Science, King Saud University, Riyadh, Saudi Arabia; ^3^Department of Podiatry, Hycare Super Speciality Hospital, MMDA Colony, Arumbakkam, Chennai, Tamil Nadu, India

## Abstract

The genetic polymorphism in the nuclear factor erythroid 2-related factor 2 (Nrf2) gene has been reported as one of the prognosis markers for various diseases, including cancer. Nrf2 is a key transcription factor involved in wound healing by regulating angiogenesis. We investigated the genetic association of *NRF2* single-nucleotide polymorphism rs35652124 with T2DM and DFU and assessed its functional impact. A total of 400 subjects were recruited for the study and categorized into three groups: infected DFU patients (DFU, *n* = 100), T2DM patients without complications (T2DM, *n* = 150), and healthy adults with normal glucose tolerance (NGT, *n* = 150). The subjects were genotyped by PCR-RFLP, and the polymorphism was identified by bidirectional Sanger sequencing. The expression of *NRF2*, *IL-10*, *TNF-α*, and *IL-6* was studied by qPCR to evaluate the functional impact of rs35652124. The “TT” genotype of rs35652124 was associated with a significant risk for T2DM [OR = 2.2 (1.2-4.2), *p* = 0.01] and DFU [OR = 7.9 (4-14.9), *p* < 0.0001]. A significant decrease in transcriptional levels of *NRF2* and *IL-10* and a remarkable increase in *TNF-α* and *IL-6* were observed in subjects with TT genotype. In conclusion, rs35652124 (TT) is a harmful genetic variant that predisposes to insulin resistance and impaired angiogenesis. Hence, it may serve as a diagnostic genetic marker for T2DM and DFU in combination with different inflammatory markers.

## 1. Introduction

Diabetic Foot Ulcer (DFU) is the fastest growing chronic complication of diabetes and a major cause of mortality in the diabetic population [1]. Amputation in subjects with diabetes is ten to twenty times higher than in subjects without diabetes [2]. The progression of DFU is often complicated by wide-ranging diabetic changes, such as neuropathy and vascular disease. Recent research is now focusing on the role of epigenetic factors, which by themselves and/or in combination with classical genetic factors, may be the major causative factor for the progression of DFU. Although genetic and epigenetic factors predispose an individual to diabetes and, these molecular mechanisms have not been completely elucidated.

Nuclear factor erythroid 2-related factor 2 (Nrf2), encoded by the gene *NRF2*, is a main redox homeostasis mediator. Nrf2 triggers an array of proteins such as glutathione-S-transferase (GST), glutathione peroxidase (GPx), UDP-glucuronosyltransferase (UGT), NAD(P)H: quinone oxidoreductase 1 (NQO1), multidrug resistance-associated protein (MRP), heme-oxygenase-1(HO-1), peroxiredoxin (Prx), Sulfiredoxin 1 (SRXN1), and Thioredoxin reductase 1 (TXNRD1) involved in cytoprotection and detoxification [3]. However, recent research has demonstrated that Nrf2 is downregulated in various inflammatory disorders [4, 5]. Studies from our laboratory have reported that circulatory levels of Nrf2 and its downstream targets were significantly low in type 2 diabetes and DFU [6, 7]. Previous investigations have reported that *NRF2* expression is regulated by genetic factors such as single-nucleotide polymorphisms (SNPs), epigenetic factors such as promoter methylation and posttranslational modifications of histones [8]. However, the molecular mechanisms that downregulate the *NRF2* expression in T2DM and DFU remain unresolved.

One of the most prevalent genetic variations that predispose an individual to diabetes and its complications is SNPs [9–12]. It induces nucleotide substitution at specific locations in a gene, causing variations in susceptibility to disease. Polymorphisms in the promoter region can regulate gene expression [13]. Epidemiological and genetic association studies have proven the association of *NRF2* promoter polymorphisms with diseases linked to oxidative stress, suggesting the genetic predisposition of *NRF2* polymorphisms to disease susceptibility [14]. Among the *NRF2* promoter polymorphisms, rs6721961, rs6706649, and rs35652124 are the most studied ones. We have chosen rs35652124 (g.178130073 C/T, c.-214 G>A) due to its involvement in regulating efficient binding of Nrf2 with promoter binding sites like antioxidant response element (ARE) [15]. Nrf2 autoregulates its activity through its ARE, and the consequence of a polymorphism in ARE could be the decline of transcriptional activity of Nrf2-dependent cytoprotective genes [16]. Previous studies have also demonstrated that rs6721961 and rs6706649 have low minor allele frequencies when compared to rs35652124 [17, 18].

The association of rs35652124 with a few diseases has been previously documented. The investigation by Córdova et al. demonstrated the association of rs35652124 with nephritis in childhood-onset systemic lupus erythematosus [19]. Zhu et al. analyzed three SNPs, namely, rs35652124, rs6706649, and rs6721961, in Hashimoto's thyroiditis and reported that the presence of one or more minor alleles was linked with a near-significant risk [20]. Collectively, these investigations demonstrated the genetic association of rs35652124 with diseases linked to autoimmunity, inflammation, and oxidative stress. However, the functional impact of rs35652124 on diabetes and DFU has never been explored. In the present study, we have analyzed the genetic association of rs35652124 with T2DM and DFU. Further, its functional impact was analyzed by measuring the expression of *NRF2*, interleukin-10 (*IL-10*), tumor necrosis factor-*α* (*TNF-α*), and interleukin-6 (*IL-6*) in the study subjects.

Our investigation demonstrated that the rs35652124 TT genotype was significantly associated with T2DM and DFU and found to have a significant decrease in transcriptional levels of *NRF2* and anti-inflammatory marker *IL-10* and a significant increase in pro-inflammatory markers *TNF-α* and *IL-6*, suggesting the inherent deleterious impacts of the polymorphism.

## 2. Patients and Methods

### 2.1. Study Population

A total of 400 participants were chosen for this cross-sectional study and grouped into three, i.e., group I: subjects with normal glucose tolerance (*n* = 150), group II: subjects with type 2 diabetes mellitus (*n* = 150), and group III: subjects with diabetic foot ulcers (*n* = 100). The research subjects were recruited from the Hycare Super Speciality Hospital, Chennai, and the blood samples were collected in the fasting state. The ethics committee of the institute approved the study protocol (025-A/HYC/IEC/2018), and all the study subjects gave written informed consent. The investigation was conducted in accordance with the Declaration of Helsinki.

### 2.2. Inclusion and Exclusion Criteria

The participants in this investigation are of south Indian origin aged 50 to 55 years. Subjects with T2DM and DFU were chosen based on the World Health Organization criteria and IDSA (Infectious Diseases Society of America)-IWDF (International Working Group on the Diabetic Foot) classification, respectively. NGT included subjects with normal FPG (72 to 99 mg/dL), PPG (below 140 mg/dL), and HbA1c (below 6.0%). T2DM subjects were identified on the basis of FPG (>100 mg/dL), PPG (200 mg/dL or more), and HbA1c (6.5% or above). Duration of diabetes was not considered. Similarly, subjects with infected DFU were selected based on their symptoms of systemic inflammatory responses (WBC >12,000 or <4000 cells/*μ*L) and wound size (≥2 cm).

Subjects with infectious diseases, peripheral vascular disease, autoimmune diseases, and haematological diseases and subjects with other reasons of harm to the peripheral nerves, such as vitamin B_12_ insufficiency, use of neurotoxic drugs, and inherited neuropathy were not considered for this investigation.

### 2.3. Anthropometric Measurements and Biochemical Parameters

Anthropometric measurements of study subjects like height and weight were obtained using standard techniques. Body mass index (BMI) calculation was done according to the formula, i.e., dividing the weight in kilograms by the height in centimeters squared. The blood pressure was measured using INFI deluxe mercury sphygmomanometer. Biochemical analysis of fasting plasma glucose (FPG), postprandial plasma glucose (PPG), total serum cholesterol, HDL-cholesterol (HDL-c), and LDL-cholesterol (LDL-c) was performed in Hitachi-912 autoanalyzer using kits supplied by Roche Diagnostics (Germany). FPG and PPG were analyzed by the glucose oxidase-peroxidase method. Total serum cholesterol was analyzed by cholesterol oxidase-peroxidase-amidopyrine method. HDL-c was measured by the direct method with polyethylene glycol-pre-treated enzymes. LDL-c was calculated by Friedewald formula. Glycated haemoglobin A1c (HbA1c) levels were analyzed using HPLC. The total blood cell counts were analysed on a hematology analyzer (XN-1000; Sysmex, Kobe, Japan).

### 2.4. Genotyping of *NRF2* rs35652124 Polymorphism

**Figure 1:**
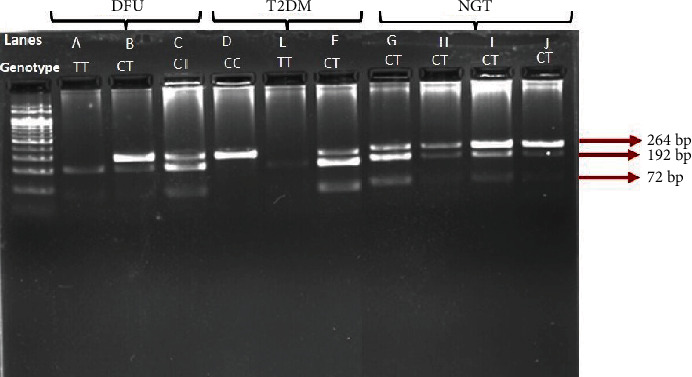
PCR-RFLP results of rs35652124. First lane shows 100 bp DNA ladder. Lanes A and E indicate the “TT” genotype with bands at 192 and 72 bp. Lane D represents CC genotype with a single band at 264 bp alone. Lanes B, C, F, G, H, I, and J indicate CT genotype with three bands at 264, 192, and 72 bps.

Genomic DNA was isolated using QIAamp DNA Mini Kit (Qiagen) according to the manufacturer's instructions. The purity and concentration of isolated DNA were analyzed using Thermo Scientific™ NanoDrop™ 2000/2000c spectrophotometer. The samples with a purity of 1.8 (A260/280) was used for the study. The *NRF2* promoter region with the polymorphism rs35652124 was amplified by S1000 thermal cycler (Bio-Rad, USA), using the primers: Forward: 5′-CCTTGCCCTGCTTTTATCTC-3′ and Reverse: 5′-CTTCTCCGTTTGCCTTTGAC-3′. The PCR was done based on the following protocol: initial denaturation of 95°C for 5 min, followed by 30 cycles of denaturation at 95°C for 30 s, annealing at 57°C for 30 s, and extension at 72°C for 1 min, followed by a final extension at 72°C for 10 min. Following PCR, the amplicon of 264 base pairs (bps) was subjected to Restriction Fragment Length Polymorphism (RFLP) using the restriction enzyme BseRI (Neb enzyme, USA) as per the manufacturer's instructions. The restriction digested products were resolved on three percent agarose gel and visualised in Syngene G Box XR5 Chemiluminescence Imaging System (Syngene International Limited, India). The homozygous wild CC genotype was obtained as 1 band (264 bp), the heterozygous CT genotype as 3 bands (264, 192, and 72 bps), and the homozygous mutant genotype TT as two bands (192 and 72 bps) (Figure 1). Further, the PCR products were purified using the QIA quick gel extraction kit (Qiagen, USA) and sequenced on SeqStudio Genetic Analyzer (Applied Biosystems, USA). The chromatograms were visualised in CodonCode Aligner (CodonCode Aligner 9.0.1).

### 2.5. Analysis of NRF2 and Inflammatory Markers by qPCR

5 mL of venous blood was obtained from the study subjects based on their rs35652124 genotypes. Further, peripheral blood mononuclear cells (PBMCs) were separated from the whole blood by a Ficoll-histopaque density gradient centrifugation method. mRNA was isolated using the RNeasy Mini Kit (Qiagen) according to the manufacturer's instructions. cDNA conversion was performed using Takara PrimeScript RT-reagent kit. The expression of *NRF2*, *IL-10*, *IL-6*, and *TNF-α* among the study subjects was analyzed by quantitative real-time PCR using CFX Connect Real-Time PCR Detection System (Bio-Rad, USA). The primers used are as follows: *NRF2* (F): 5′-TGTAGATGACAATGAGGTTTC-3′, *NRF2* (R): 5′-ACTGAGCCTGATTAGTAGCAA-3′*, IL-10* (F): 5′-ACATCAGGGTGGCGACTCTA-3′, *IL-10* (R): 5′-AAGGTTTCTCAAGGGGCTGG-3′, *IL-6* (F): 5′-GTCCAGTTGCCTTCTCCCTG-3′, *IL-6* (R): 5′-AGCACGACCACGACCTTG-3′, *TNF-α* (F): 5′-TCTGGGCAGGTCTACTTTGG-′, *TNF-α* (R): 5′-GGTTGAGGGTGTCTGAAGGA-3′, *GAPDH* (F): 5′-AAGAAGGTGGTGAAGCAGGC-3′, and *GAPDH* (R): 5′-GTCAAAGGTGGAGGAGTGGG-3′.

### 2.6. Statistical Analysis

Statistical analysis was carried out using the SPSS version 20.0. The data of continuous variables are represented as mean ± SD. For non-normally distributed variables, Mann-Whitney *U* tests were used to compare medians. The analysis of Hardy Weinberg equilibrium was performed by chi-square test. Determination of frequencies of alleles and genotype distribution were performed by chi-square test of independence with two by twofold contingency and *z*-score. Multivariate logistic regression analysis calculated the odds ratio (OR) with ninety-five percent confidence interval. *p* values of less than 0.05 were regarded as statistically significant.

## 3. Results

### 3.1. Biochemical Characteristics of the Study Subjects

The clinical and biochemical characteristics such as blood glucose, blood pressure, lipid profile, and HOMA index of the study subjects are depicted in Table 1. The NGT subjects were normoglycemic with BMI and biochemical parameters in the normal range. Reflecting the severity of the disease, SBP, DBP, FPG, PPG, HbA1c, HOMA-IR, LDL-c, total serum cholesterol, and WBC counts were significantly high among T2DM and DFU subjects when compared to NGT (*p* < 0.001), whereas HDL-c levels were significantly low in T2DM and DFU when compared to NGT (*p* < 0.001).

### 3.2. The nsSNP rs35652124 (g.178130073T>C) Is Associated with T2DM and DFU

**Figure 2:**
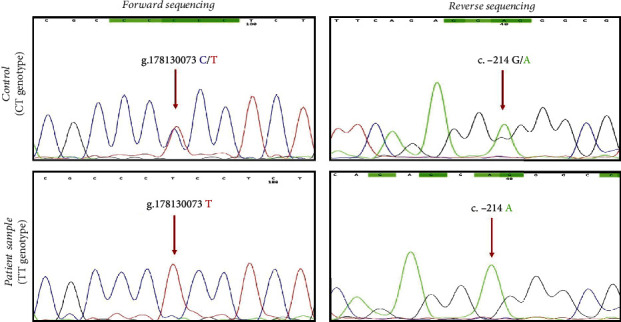
Bidirectional Sanger sequencing results of rs35652124. In forward sequencing, “C” peak is indicative of wild allele, and “T” peak is indicative of mutant allele. Similarly, in reverse sequencing, “G” peak is indicative of wild allele, and “A” peak is indicative of mutant allele.

Table 2 depicts the genetic interrelation of rs35652124 with T2DM and DFU. The frequency of TT genotype was significantly higher in DFU subjects (52%) when compared to T2DM (23.3%) and NGT subjects (12%). Patients with TT genotype had a significant risk for the pathogenesis of T2DM and DFU when compared against NGT vs. T2DM (OR = 2.2 (1.2-4.2), *p* = 0.01) and NGT vs. DFU (OR = 7.9 (4-14.9), *p* < 0.0001), relative to subjects with CT and CC genotypes. The minor allele ‘T' was significantly prevalent among the DFU subjects relative to T2DM (2.2 (1.5-3.2), *p* = 0.0001) and NGT subjects (2.9 (2-4.3), *p* < 0.0001). As depicted in Figure 2, bidirectional Sanger sequencing identified polymorphism in the study subjects. In forward sequencing, “T” peak is indicative of mutant allele, and “C” peak is indicative of wild allele. Similarly, in reverse sequencing, “A” peak is indicative of mutant allele, and “G” peak is indicative of wild allele.

### 3.3. Low NRF2 in T2DM and DFU Patients with rs35652124 (TT) Genotype

**Figure 3:**
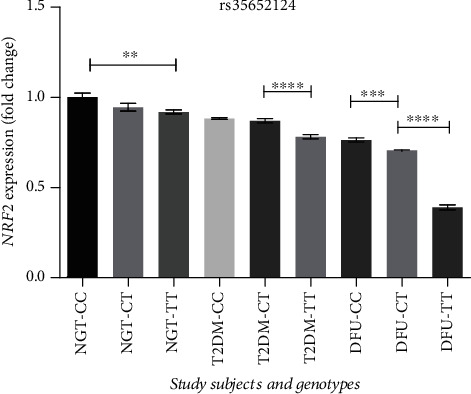
Relative gene expression of *NRF2* in PBMCs of study subjects based on their genotype. All data are reported as mean ± SEM; ^∗∗∗∗^*p* < 0.0001, ^∗∗∗^*p* < 0.001, ^∗∗^*p* < 0.01.

As depicted in Figure 3, *NRF2* expression was significantly downregulated in DFU subjects when compared to T2DM and NGT subjects. But analysis of *NRF2* expression based on patient's genotype demonstrated that DFU (2-fold, *p* < 0.0001), T2DM (1.1-fold, *p* < 0.0001), and NGT (1-fold, *p* < 0.001) subjects with the homozygous mutant TT genotype had a greater decline in *NRF2* expression when compared to T2DM and DFU subjects with homozygous wild CC genotype. This is suggestive of the inhibitory effect of TT genotype in suppressing *NRF2* expression.

### 3.4. Dysregulation in Transcriptional Levels of Pro-inflammatory and Anti-inflammatory Markers in T2DM and DFU Patients with rs35652124 (TT) Genotype

**Figure 4:**
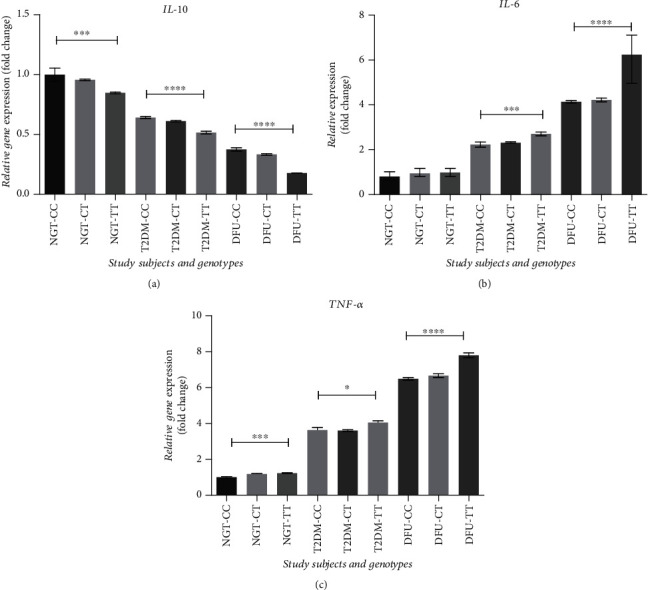
Relative gene expression of (a) *IL-10*, (b) *IL-6*, and (c) *TNF-α* in the study cohorts based on genotype. All data are reported as mean ± SEM; ^∗∗∗∗^*p* < 0.0001, ^∗∗∗^*p* < 0.001, ^∗^*p* < 0.05.

The transcriptional levels of anti-inflammatory cytokine *IL-10* and pro-inflammatory cytokines *IL-6* and *TNF-α* were analyzed in study subjects based on their genotype. As depicted in Figure 4, DFU subjects with the homozygous mutant TT genotype were observed to have a significant increase in *IL-6* and *TNF-α* expression when compared to T2DM and DFU subjects with homozygous wild CC genotype, whereas *IL-10* expression was significantly decreased in DFU subjects with the homozygous mutant TT genotype when compared to T2DM and DFU subjects with homozygous wild CC genotype.

**Figure 5:**
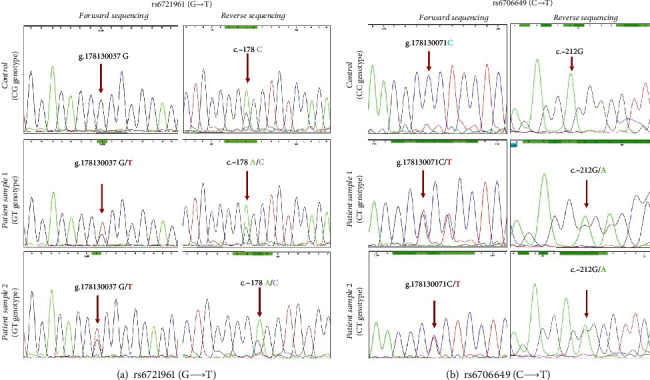
Bidirectional Sanger sequencing results of (a) rs6721961: in forward sequencing, “G” peak indicates the wild allele, and “T” peak indicates the mutant allele. Similarly, in reverse sequencing, “C” peak indicates wild allele, and “A” peak indicates mutant allele; (b) rs6706649: in forward sequencing, “C” peak indicates the wild allele, and “T” peak indicates the mutant allele. Similarly, in reverse sequencing, “G” peak indicates the wild allele, and “A” peak indicates the mutant allele.

Further, to understand the influence of the other two well-known SNPs in *NRF2* promoter, namely, rs6721961 (G>T) and rs6706649 (C>T) on *NRF2* regulation, we performed bidirectional Sanger sequencing in NGT and DFU subjects. As represented in Figure 5, none of the analyzed subjects had homozygous mutant genotype at rs6721961 and rs6706649 loci. NGT subjects were observed to have homozygous wild genotype at rs6721961 and rs6706649 loci, whereas DFU subjects were observed to have heterozygous genotype. However, in-depth genetic association studies are required to gain more evidence.

## 4. Discussion

Prolonged hyperglycemia and cellular oxidative stress are the prime pathophysiological determinants of DFU [21]. In healthy subjects, the oxidative stress is counteracted by efficient cellular antioxidant machinery. But in diabetic subjects, prolonged hyperglycemia and oxidative stress result in the generation of excessive reactive oxygen species which causes endothelial dysfunction, vascular damage, and delayed wound healing [22]. Nrf2 is a transcription factor that maintains redox homeostasis in cells. It grants endogenous cellular security to cells by activating antioxidant and detoxifying genes that resist cellular stress [23]. Studies have confirmed the downregulation of Nrf2 in several diseases including diabetes [24, 25]. Extensive investigations indicate that the Nrf2-Keap1 cascade performs a pivotal role in redox homeostasis [26].

Under normal physiology, Nrf2 is subjected to proteasomal degradation by its negative regulator Keap1. However, during cellular stress, the Keap1 cysteine residues get covalently modified, and this enables Nrf2 to dissociate from Keap1 complex, moved to the nucleus, and transcribes an array of genes responsible for detoxification and antioxidant mechanism. Hence, the Nrf2-Keap1 complex acts as a sensor for redox status [27]. However, the genetic mechanisms behind the dysregulation of *NRF2* expression in T2DM and DFU remain unexplored.

Downregulation of Nrf2 is one of the significant factors that cause impaired angiogenesis in DFU subjects [28]. The dysfunction of the *NRF2* by SNPs is gradually becoming a milestone to discern disease development and progression in diabetes [29]. The presence of SNPs in the regulatory motifs of *NRF2* gene would affect the efficient binding of transcription factors to the gene and consequently repress transcription [30]. The present study analyzed the *NRF2* SNP rs35652124 (g.178130073 C/T, c.-214 G>A), in T2DM and DFU subjects. This polymorphism is located at position -214 of the *NRF2* promoter and adversely affects the binding of Nrf2 to antioxidant response element (ARE) like promoter binding sites [15].

The present study demonstrated that *NRF2* rs35652124 “TT” genotype was remarkably higher in T2DM and DFU subjects than in healthy control and conferred significant risk for the pathogenesis of T2DM and DFU. In addition, it demonstrated that *NRF2* expression was significantly decreased in DFU patients with TT genotype. These observations have coincided with the report of Santos et al., which demonstrated that rs35652124 was associated with a lower level of *NRF2* expression in a cohort of alcoholic liver disease subjects [31]. Besides, a few previous studies have also demonstrated that SNP rs35652124 (TT) decreases the binding of Nrf2 to ARE and hence downregulates the transcriptional activity of Nrf2 [15, 32].

Investigations by Shimoyama et al. have shown that *NRF2* rs35652124 TT genotype is associated with increased risk for blood pressure in a Japanese cohort [33]. Shimoyama et al. have also demonstrated that TT genotype is a strong predisposing risk factor for cardiovascular mortality in Japanese hemodialysis patients [34]. Similarly, Song et al. have also demonstrated that *NRF2* rs35652124 TT SNP confers vitiligo risk in Han Chinese subjects [32]. All these previous investigations support our findings and confirm that *NRF2* rs35652124 TT is a harmful genetic variant of *NRF2*.

In contrast, there are a few investigations that suggested that *NRF2* rs35652124 TT genotype is not a harmful genetic variant, whereas *NRF2* rs35652124 CC genotype is harmful. For example, based on studies of Marczak et al. in African Americans, subjects with “C” variant allele had significantly lower forearm blood flow and higher forearm vascular resistance when compared to healthy control. Besides, following oxidative stress, the C allele exhibited reduced *NRF2* expression compared with the “T” allele. The disparity in these findings with the present study could be mainly due to differences in ethnicity [35]. The present study was conducted in the South Indian population, whereas the aforementioned study was conducted in African Americans. Likely, rs35652124 SNP acts differently in diverse ethnic population, and samples according to which transcription factors are regulated [17].

Further, to validate the functional impacts of the rs35652124 TT genotype on insulin resistance and wound healing process, we have studied the transcriptional profile of *IL-10*, *TNF-α*, and *IL-6* in the study cohort based on their genotype. The decline of *IL-10* and elevation of *TNF-α* and *IL-6* are reported to be important regulators underlying insulin resistance and a slow nonhealing chronic wound process [36, 37]. Consistent with these findings, we found a reduced expression of *IL-10* and a significant increase in *TNF-α* and *IL-6*, in T2DM and DFU subjects with TT genotype. In summary, these findings confirm that the *NRF2* rs35652124 TT genotype dysregulate key genes involved in redox homeostasis and wound healing, and hence, it is a predisposing risk factor in the pathogenesis of T2DM and DFU.

This work represents an advance in biomedical science as it demonstrates that *NRF2* SNP rs35652124 serves as a diagnostic genetic marker for T2DM and DFU in the South Indian population and is associated with decreased transcriptional levels of *NRF2* in these subjects. There are no investigations, so far, representing the clinical significance of this polymorphism in T2DM and DFU. The strength of this case-control study is that it was confined to subjects with the same ethnicity. However, the shortcoming of the study is its cross-sectional nature, which implies that no cause and effect relationship can be conveyed. Further studies are required in a diverse ethnic population to gather more evidence.

## Figures and Tables

**Table 1:** Clinical and biochemical characteristics of the study population.

Clinical parameters	NGT (*n* = 150)	T2DM (*n* = 150)^a^	DFU (*n* = 100)^b^
Gender (M/F)	77M/73F	79M/71F	48M/52F
Age (years)	51.6 ± 1.2	51.4 ± 1.4	52.8 ± 1.6
BMI (kg/m^2^)	21.2 ± 1.6	27.6 ± 1.4^∗∗∗∗^	28.1 ± 1.7^∗∗∗∗^
SPG (mmHg)	102.2 ± 6.9	118.6 ± 7.8^∗∗∗∗^	128.5 ± 3.5^∗∗∗∗^
DBP (mmHg)	75.3 ± 4.1	81.1 ± 3.2^∗∗∗∗^	86.3 ± 2.5^∗∗∗∗^
FPG (mg/dL)	89.9 ± 7.2	136.6 ± 8.5^∗∗∗∗^	215.2 ± 11.4^∗∗∗∗^
PPG (mg/dL)	100.9 ± 11.2	234.5 ± 10.3^∗∗∗∗^	271.2 ± 39.7^∗∗∗∗^
HbA1c (%)	5 ± 0.6	9.4 ± 0.5^∗∗∗∗^	11 ± 0.9^∗∗∗∗^
Total serum cholesterol (mg/dL)	128.4 ± 22.4	139.9 ± 22.4^∗∗∗∗^	190 ± 5^∗∗∗∗^
HDL-cholesterol (mg/dL)	60.3 ± 4.1	47.6 ± 4.2^∗∗∗∗^	43.5 ± 1.9^∗∗∗∗^
LDL-cholesterol (mg/dL)	94.7 ± 7.6	108.8 ± 9.3^∗∗∗∗^	124.3 ± 4.5^∗∗∗∗^
HOMA-IR	1.3 ± 0.3	3.2 ± 1.1^∗∗∗∗^	7.4 ± 1^∗∗∗∗^
WBC count (10^9^/L)	6.3 ± 1.5	8.1 ± 1.5^∗∗∗∗^	13.4 ± 2.5^∗∗∗∗^

All data are reported as mean ± SD for continuous variables; ^∗∗∗∗^*p* < 0.0001; ^a^comparison between T2DM and NGT; ^b^comparison between T2DM and DFU.

**Table 2:** Distribution of allele and genotype frequencies and genetic interrelation of rs35652124 SNP with T2DM and DFU: odds ratio (OR) for minor alleles and their homozygous and heterozygous genotypes.

rs35652124	Genotypes			Alleles	
	CC	CT	TT	C	T
NGT	27 (18%)	105(70%)	18 (12%)	159(53%)	141 (47%)
T2DM	22(14.7%)	93(62%)	35(23.3%)	137(46%)	163 (54%)
DFU	8(8%)	40(40%)	52(52%)	56(28%)	144(72%)
NGT vs. T2DM OR (95% CI)	0.8 (0.4-1.4) *p* = 0.4	0.7 (0.4-1.1) *p* = 0.14	**2.2 (1.2-4.2)** **p** = 0.01	0.5 (0.3-0.7) *p* = 0.0001	1.3 (1-1.8)*p* = 0.07
NGT vs. DFU OR (95% CI)	0.4 (0.2-0.9) *p* = 0.03	0.3 (0.2-0.5) *p* < 0.0001	**7.9 (4-14.9)** **p** < 0.0001	0.3 (0.2-0.5) *p* < 0.0001	**2.9 (2-4.3)** **p** < 0.0001
T2DM vs. DFU OR (95% CI)	0.5 (0.2-1.2) *p* = 0.12	0.4 (0.2-0.7) *p* = 0.0007	**4 (2.1-6.1)** **p** < 0.0001	0.5 (0.3-0.7) *p* = 0.0005	**2.2 (1.5-3.2)** **p** = 0.0001

Figures in bold are significant with *p* < 0.05 and has odds ratio greater than one, suggesting the pathogenicity.

## Data Availability

The data used to support the findings of this study are available from the corresponding author upon request.
